# Supplementary material for: Tracking Smell Loss to Identify Healthcare Workers with SARS-CoV-2 Infection
Source: medRxiv. 2020 Sep 10:2020.09.07.20188813. Preprint. [Version 2] doi: 10.1101/2020.09.07.20188813 (PMC7491536; doi:10.1101/2020.09.07.20188813)
Supplement: Supplement 2020 [file 96043-2020.09.07.20188813-1.docx]

**Supplemental Material**

**Supplemental Table 1. The Yale Jiffy Survey**

| **Initial questions** | **Answer options** |  |
| --- | --- | --- |
| How would you rate your ability to smell | Poor, average, good, very good |  |
| Have you noticed a reduction in your sense of smell in the past week? | None, slight, moderate, severe |  |
| Please indicate how much reduction (if any) you believe you have experienced in the past week | 100mm scale (“no reduction at all” to “extreme reduction”) |  |
| **Smell test instructions** | **Smell test questions** | **Smell test answer options** |
| Please find a jar of peanut butter (or other nut butter). If you have any nut allergies, please use jam or jelly. | What do you have? | Peanut butter, other nut butter, jelly, jam, other |
| Open it and bring it to about 1 inch from your nose. Please sniff. | Do you smell it? | Yes/No |
|  | How strong does it smell? | 100mm scale (“no sensation” to “strongest sensation imaginable”) |
|  | Does it smell different from normal? | Yes/No |
|  | Please indicate how different it is | 100mm scale (“no different” to “completely different”) |
| Please find a jar of vinegar (white vinegar is best) or a muscle balm (e.g., Tiger balm or Bengay). | What do you have? | Vinegar, Tiger balm, Bengay, nail polish remover, rubbing alcohol, other |
| Open it and bring it to about 1 inch from your nose. Please sniff. | Do you feel a sensation of irritation (e.g., burning, stinging, harshness) in your nose or throat? | Yes/No |
|  | How strong was the sensation? | 100mm scale (“no irritation” to “strongest irritation imaginable”) |
|  | Does it smell different from normal? | Yes/No |
|  | Please indicate how different it is | 100mm scale (“no different” to “completely different”) |

**Supplemental Table 2.** **Parosmia Questionnaire**

| How often are you bothered by any of the below? | Always | Often | Rarely | Never |
| --- | --- | --- | --- | --- |
| 1. Food tastes different than it should because of a problem with odors. | 1 | 2 | 3 | 4 |
| 2. I always have a bad smell in my nose, even if no odor source is present. | 1 | 2 | 3 | 4 |
| 3. Odors that are pleasant to others are unpleasant to me. | 1 | 2 | 3 | 4 |
| 4. The biggest problem is not that I do not or only weakly perceive odors, but that they smell different than they should. | 1 | 2 | 3 | 4 |

Adapted from Landis BN, Frasnelli J, Croy I, Hummel T. Evaluating the clinical usefulness of structured questions in parosmia assessment. *Laryngoscope*. 2010;120(8):1708.

**Supplementary Table 3.**  **Characteristics of IMPACT Study HCW Who Were Included and Excluded from the Smell Sub-Study.**

|  | IMPACT HCW Included in Smell Sub-Study  (*n* = 473) | IMPACT HCW Excluded from Smell Sub-Study  (*n* = 115) | *P* Value^a^ |
| --- | --- | --- | --- |
| Demographics |  |  |  |
| Age, y | 34.0 (29.0, 44.0) | 34.0 (28.0, 42.0) | 0.62 |
| Female sex | 374 (79) | 91 (79) | 1 |
| Ethnicity |  |  | <0.001 |
| White | 375 (79) | 67 (58) |  |
| Black | 15 (3) | 18 (16) |  |
| Hispanic | 37 (8) | 8 (7) |  |
| Asian | 37 (8) | 14 (12) |  |
| Other | 9 (2) | 8 (7) |  |
| BMI, kg/m^2^ | 24.7 (22.7, 29.1) | 27.1 (23.4, 31.6) | 0.004 |
| Occupation |  |  | 1 |
| RN | 261 (55) | 64 (56) |  |
| MD | 97 (21) | 23 (20) |  |
| Other | 115 (24) | 28 (24) |  |

Data are presented as median (IQR) for continuous variables and no. (%) for categorical variables.

Abbreviations: BMI, body mass index; HCW, healthcare workers; IMPACT, Implementing Medical and Public Health Action against Coronavirus (CT); MD, medical doctor; RN, registered nurse.

^a^ Unadjusted *P* values are Wilcoxon rank sum test (continuous variables) or Fisher’s exact test (categorical variables).

**Supplementary Figure Caption**

**Supplementary Figure 1.** Results of matching on age, sex, profession, ethnicity, and number of symptom questionnaires completed for HCW reporting smell on either survey (Panels a & b), the daily symptom survey only (Panels c & d), and the Yale Jiffy only (Panels e & f). Panels a, c, & e show the change an absolute standardized difference in means between the unmatched (“All Data”) and matched datasets. Panels b, d, & f compare the distributions, as histograms, of the propensity scores between HCW with smell loss (“Treated”) and those without (“Control”) both before (“Raw”) and after (“Matched”) matching. In all cases, matching resulted in a decrease in the absolute standardized difference for the overall distance measure and a more similar distribution (as shown by histograms) of propensity scores.
