# Supplementary figures and images for: Tracking Smell Loss to Identify Healthcare Workers with SARS-CoV-2 Infection
Source: medRxiv. 2020 Sep 10:2020.09.07.20188813. Preprint. [Version 2] doi: 10.1101/2020.09.07.20188813 (PMC7491536; doi:10.1101/2020.09.07.20188813)

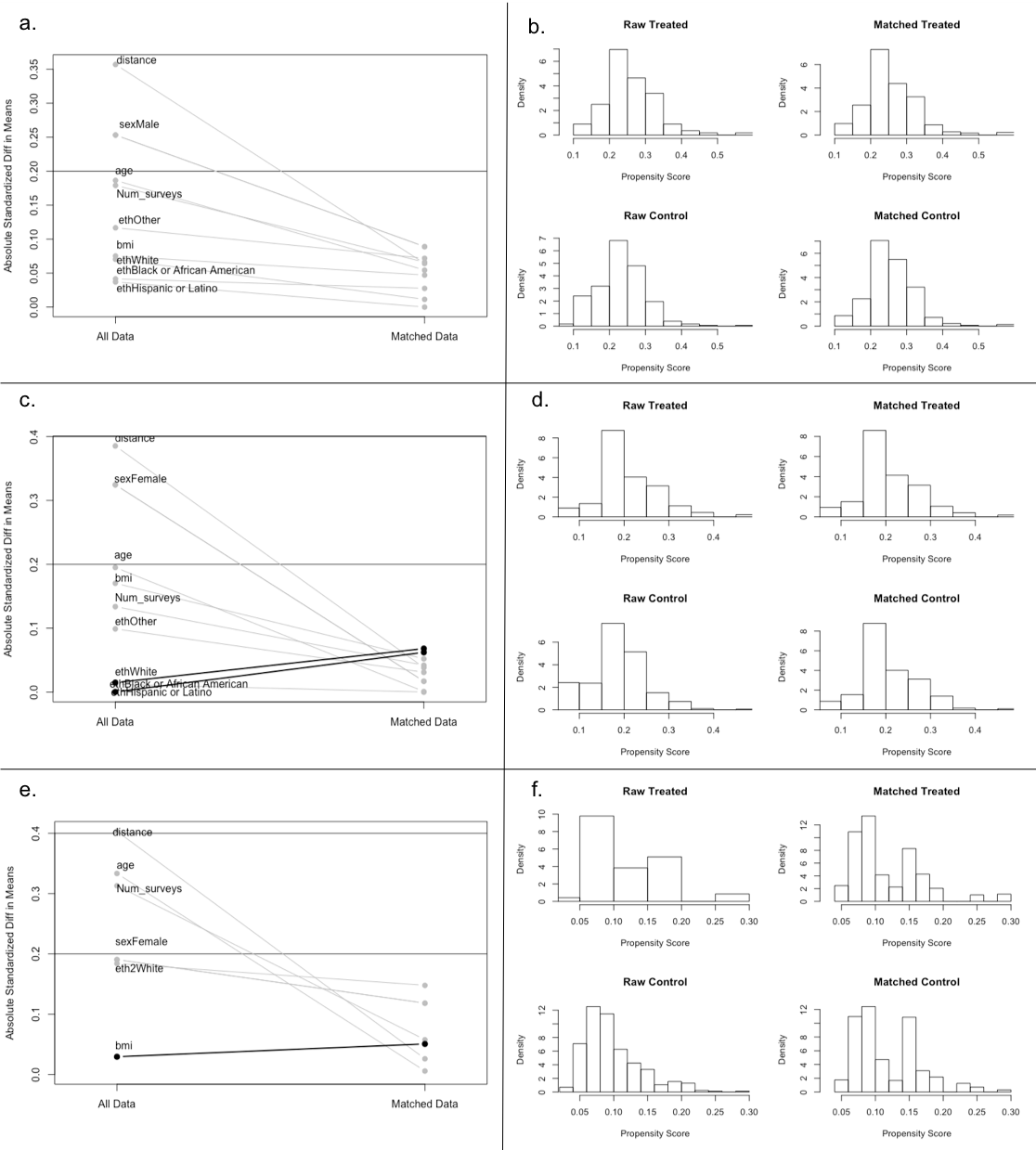

Supplement: Supplement 2020 [file 96043-2020.09.07.20188813-2.tif]
